# Metabolomic Prediction of Naphthalene Pneumo-Toxicity in the Snail *Helix aspersa maxima*

**DOI:** 10.3390/metabo15070448

**Published:** 2025-07-03

**Authors:** Aude Devalckeneer, Marion Bouviez, Jean-Marie Colet

**Affiliations:** Laboratory of Human Biology & Toxicology, Faculty of Medicine and Pharmacy, University of Mons, 7000 Mons, Belgium; aude.devalckeneer@umons.ac.be (A.D.);

**Keywords:** ^1^H-NMR, respiratory toxicity, snails, naphthalene, biomarkers

## Abstract

**Background:** Polluted soils represent a major problem in many industrialized countries that urgently requires appropriate health risk assessment. The One Health concept that considers a close relationship between human and animal health and ecosystems relies, among other techniques, on continuous monitoring through the use of animal species as bioindicators. In this context, terrestrial gastropods, already recognized as relevant indicators due to their anatomo-physiology, provide a reliable model to study the pneumotoxic effects of pollutants. On the other hand, risk assessment is based on multi-biomarker studies. Therefore, omic approaches seem particularly useful since they can simultaneously detect numerous early biological changes. **Methods:** In this study, *Helix aspersa maxima* was exposed to naphthalene, a highly volatile aromatic hydrocarbon responsible for numerous respiratory disorders. Pulmonary membrane extracts and hemolymph samples were analyzed by ^1^H-NMR spectroscopy after single or repeated exposures to naphthalene. **Results:** Numerous metabolic changes were observed, which could be related to membrane lesions, energy, anti-inflammatory, and tumorigenesis pathways. **Conclusions:** Our findings highlight the potential of combining animal indicator and omics techniques to predict respiratory health risks in cases of exposure to polluted soils.

## 1. Introduction

Anthropic pollution is a major public health issue in many industrialized countries, requesting soil restoration and continuous health risk assessment. Contaminated soils can impact all living organisms via various exposure routes, including inhalation of volatile compounds such as polycyclic aromatic hydrocarbons (PAHs). The “One Health Concept” considers a close relationship between human and animal health and ecosystems, showing an interest in combining human health and environmental risk assessments. Therefore, it is more and more accepted that the management of polluted soils should include animal species as bioindicators to anticipate human health risks.

For more than thirty years, gastropod mollusks have been recognized as relevant ecological indicators [1,2,3,4,5,6] because they represent a significant biomass of the soil community and have a global distribution [7]. Additionally, they integrate multiple sources and pathways of contamination thanks to their presence in the soil–plant–air interface [8]; they have significant bioaccumulation capacities for many pollutants, whether of metallic [9,10,11] or organic origin [12,13]; they exhibit physiological and biochemical responses upon exposure to xenobiotics [12,14,15]; and finally, they constitute a key element of the food chain reflecting the transfer of pollutants within trophic networks [16,17,18]. Land snails, such as *Helix aspersa maxima* (Taylor, 1883) [19], classified as pulmonated organisms, have a vascularized epithelium acting as a lung, lining the mantle cavity and receiving oxygen via the pneumostome. As such, they represent an appropriate model to assess respiratory risks of exposure to atmospheric pollutants.

Nowadays, effective health risk monitoring using a bioindicator model is expected to be based on a multi-biomarker approach [20,21,22]. In this context, ^1^H-NMR- and MS-based metabolomics can give a simultaneous and global assessment of the alterations in the metabolome in one single analysis. It is also seen as one of the few techniques able to help transpose animal findings to humans since most of the cellular biochemical pathways are conserved through evolution [23,24], one major goal of the “One Health Concept”. Recent studies have shown the advantages of using the ^1^H-NMR-based metabolomics approach in air pollution research through the exploration of metabolic alterations in various biofluids [25,26], including bronchial alveolar lavage fluid (BALF) [27,28]. Such protocols allowed the early detection of metabolic changes mirroring increased oxidative stress, airway inflammation, and endothelial dysfunction.

Naphthalene, one of the carcinogenic PAHs to which humans are most exposed through various sources, including industrial waste, vehicle emissions, or tobacco smoking, is known to cause histological damages in airway epithelia associated with Clara cell necrosis [29,30,31,32]. Mechanistically, the toxicological properties of naphthalene are directly attributed to cytotoxicity and indirectly to genotoxicity after liver bioactivation and distribution of the reactive metabolites to the lungs via the systemic bloodstream [33,34,35]. After repeated and/or long-term exposures to naphthalene, naphthalene-induced cell necrosis can lead to tumor development in mammals. Interestingly, tumorigenesis is a pathological process also encountered in mollusks [36,37,38,39,40],

A couple of studies using ^1^H-NMR spectroscopy have already monitored naphthalene-induced biochemical changes in the respiratory system of rodents after intraperitoneal exposure [41,42]. Interestingly, some tolerance against naphthalene after repeated exposures was reported in mice, underlying the important role of the glutathione S-transferase (GST) enzyme in the detoxifying process [43]. GSTs are phase II conjugation enzymes in xenobiotic metabolism whose main role is to conjugate GSH to compounds containing an electrophilic moiety, as is the case in PAHs, and are found in many invertebrates such as terrestrial mollusks [33,44,45,46,47].

In this study, we monitored by ^1^H-NMR spectroscopy the changes induced in the bioindicator snail model after single and repeated exposures to naphthalene, with a special focus on early metabolic markers of respiratory damage and tumorigenesis. GST measurement was used as an indicator of naphthalene exposure and metabolism.

## 2. Materials and Methods

### 2.1. Animal Housing

*Helix aspersa maxima* individuals purchased from the helicicole farm of Avesnois (**Croix-Caluyau**, France) were housed in a homemade breeding housing, allowing permanent access to water and professional food farming provided by Helinove product (France). They were kept at a temperature of 20 ± 2 °C and daylighting. Only adults with close age and body weight (~20 g) were investigated. A total of 36 snails were used in this study, with 6 individuals per group.

### 2.2. Chemicals

The following compounds were used for the preparation and analysis of the samples: phosphate buffer–D_2_O prepared with Na_2_HPO_4_·2H_2_O 0.2 M and NaH_2_PO_4_.H_2_O 0.04 M in distilled water–deuterium oxide (D_2_O) solution (80:20, v:v); methanol; chloroform; TSP (deuterated sodium 3-trimethylsilyl propionate); DMSO (dimethyl sulfoxide 99,5%); phosphate buffer–EDTA prepared with Na_2_HPO_4_.2H_2_O 0.2 M, NaH_2_PO_4_.H_2_O 0.04 M, and EDTA (ethylene diamine tetra-acetic acid) 1 mM in distilled water; dye reagent concentrate for protein assay (5000006 from BIORAD, Hercules, CA, USA); bovine serum albumin (BSA); glutathione S-transferase assay kit (CS0410 from Sigma Aldrich, Saint Louis, MO, USA); corn oil; and naphthalene (147141-25G, 99% from Sigma Aldrich BVBA, Overijse, Belgium).

### 2.3. Naphthalene Exposure

Snails were exposed to a dose of 200 mg/kg of naphthalene. This dose was selected based on data published in rodents, for which an intramuscular dose of 200 mg/kg was most frequently used in respiratory toxicity studies [30,32,41,42,48]. More precisely, *Helix aspersa maxima* individuals received an intramuscular injection of 200 mg/kg of naphthalene, corresponding to 4 mg for a 20 g weighted snail. Injections were applied in the ¾ posterior part of the foot to minimize the risk of damaging any vital body organ with the needle while allowing a systemic distribution of the test compound after its absorption in the hemolymph according to an internal procedure described elsewhere (Devalckeneer et al., 2024) [49]. Three exposure groups with a minimum of 12 snails each were constituted as follows: in the “24 h Group”, animals were exposed to one single dose of naphthalene during 24 h and then euthanized; in the “4 days Group”, the individuals were exposed to one shot of naphthalene and then euthanized after 4 days; and finally, in the “Daily Group”, the individuals received a daily dose of naphthalene for 4 consecutive days before euthanasia. Hemolymph was sampled after 24 h and 4 days of daily exposure, and 6 snails from each group were euthanized by freezing them at −20 °C. Snails were dissected on ice to sample the pulmonary membrane. Tissue samples were immediately frozen in liquid nitrogen and stored at −80 °C until further GST activity measurements (*n* = 6 per group) and ^1^H-NMR spectroscopy analysis (*n* = 6 per group).

Control individuals (*n* = 6 per group) were exposed to corn oil, the vehicle used for the injection of naphthalene-containing solutions, following the same protocol. For each tested group, a matching control group was used.

### 2.4. Hemolymph Sampling

The procedure used in this study is based on the validated protocol previously described in Cooper (1993) [50].

Briefly, snails were washed in cold water to remove feces and excess mucus before hemolymph sampling. Snails were placed upside down, and a 25 G needle was inserted just below the pneumostome to allow hemolymph to flow out. A volume of 500 µL of a pale blue-colored liquid corresponding to hemolymph was collected in Eppendorf tubes. Amicon^®^ Ultra-0.5 Centrifugal Filter Devices (Millipore, Burlington, MA, USA) were rinsed four times with 500 μL of demineralized water and centrifuged for 15 min at 14,000× *g*. Then, 500 µL of filtered hemolymph was centrifuged (30 min at 14,000× *g*) before adding 250 µL D_2_O and centrifuged again (30 min at 14,000× *g*). Filtrates were kept at −80 °C until ^1^H-NMR analysis.

### 2.5. Measurement of GST Activity

The procedure used in this study was based on a commercially available colorimetric GST assay kit CS0410 (Sigma Aldrich, BVBA, Overijse, Belgium) using the conjugation of the thiol group of glutathione to the 1-chloro-2,4-dinitrobenzene (CDNB) substrate.

#### 2.5.1. Sample Preparation

A sample of 200 mg of pulmonary membrane, kept at −80 °C, was crushed into powder in a mortar with liquid nitrogen and extracted with 2 mL of cold 0.1 M phosphate buffer with EDTA. The mixture was then vortexed and sonicated for 40 s (6% amplitude) at 4 °C before centrifugation (30 min at 1500× *g*, 4 °C). The supernatant was collected and centrifuged (15 min at 4000× *g*, 4 °C) to repeat the procedure with a final centrifugation of the supernatant (10 min at 10,000× *g*). This final supernatant, corresponding to the cytosolic fraction, was used to measure the GST activity.

#### 2.5.2. GST Assay

A volume of 4 µL of prepared pulmonary sample was mixed with 196 µL of reaction solution (GSH 200 mM, CDNB 100 mM in Dulbecco buffer) just before the measurement at the onset of the reaction. Blank was measured with 200 µL of reaction solution. All measurements were performed in triplicates at 380 nm and recorded every minute for 15 min (FLUOstar OPTIMA spectrophotometer). The net GST activity was calculated by subtracting the non-enzymatic reaction given by the blank sample and the kinetic activity represented by the slope of the curve. This net activity was converted into specific enzymatic activity by considering the extinction molar coefficient of the thiol group and volumes used. The results were normalized against the protein content given by the Bradford method with bovine serum albumin (BSA) as standard. Statistical analysis was performed using a Mann–Whitney–Wilcoxon test using R software. version 4.5.0.

### 2.6. ^1^H-NMR Spectroscopy and Spectral Data Analysis

The procedure of sample preparation and ^1^H-NMR spectroscopy on tissue extracts used in this study is based on previous studies of *Helix aspersa maxima* tissues [51].

#### 2.6.1. Sample Preparation and Acquisition of Metabolic Profiles

Pulmonary membrane tissue samples preserved at −80 °C were crushed in a mortar with liquid nitrogen into powder and were extracted with precooled solutions according to the methanol–water–chloroform extraction method (1:1:0.8 *v*/*v*). The top aqueous phase was recovered, and methanol was removed in vacuo for 6 h. Each extract was reconstituted in 700 µL of phosphate buffer–D_2_O 0.1 M, and 50 µL of TSP (D_4_-trimethylsilyl propionic acid) 7 mM was added to 650 µL supernatant after final centrifugation. Regarding hemolymph samples, 600 µL of previously filtered preparations were mixed with 100 µL of TSP 4 mM. TSP prepared in 100% D_2_O was used as an external reference for the calibration of the NMR spectra. The singlet resonance arising from the -CH_3_ groups in TSP was arbitrarily fixed at 0.00 ppm for further spectral calibration. Finally, 700 µL of each sample was transferred into individual tubes (5 mm diameter) for NMR analysis.

One-dimensional NMR spectra of extracts were acquired on a Bruker (Billerica, MA, USA) 600 Avance spectrometer (11,8 T corresponding to a proton Larmor frequency of 600 MHz) at 297 K using a NOESYPRESAT-1d pulse sequence. A total of 128 free induction decays (FIDs) with 65.536 data points per FID were collected for tissue extract using a spectral width of 10.330,578 Hz, an acquisition time of 3.17 s, and a pulse recycle delay of 3 s.

#### 2.6.2. Statistical Analysis

Discriminant metabolites were extracted with the variable importance in projection (VIP) list of the supervised PLS-DA model (partial least squares discriminant analysis), which identifies those with a cut-off VIP score ≥ 1 as mainly contributing to the clustering. A Mann–Whitney–Wilcoxon statistical test was applied to the AUC (area under the curve) values on those VIPs to assess the significance of metabolites responsible for intergroup differences (*p*-value < 0.05 significance level) between the naphthalene exposure group and the corresponding control group. The non-parametric Wilcoxon statistical test was chosen considering the semi-quantitative data provided by AUC values.

## 3. Results

### 3.1. GST Activity in Pulmonary Membrane After Exposure to Naphthalene

**Figure 1:**
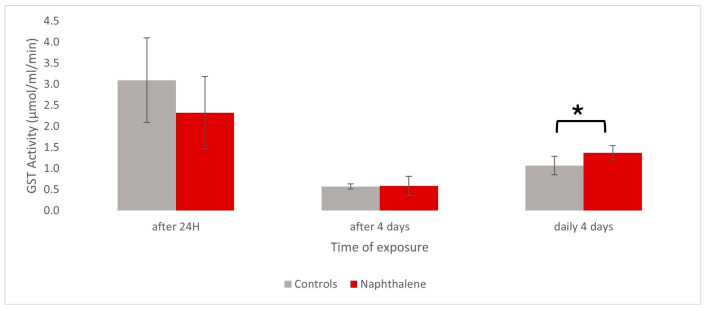
Illustration of the in vivo effect of naphthalene (4 mg/20 g snail) on specific GST activity in pulmonary membrane of *H. aspersa maxima* snails (*n*= 6) after 24 h, 4 days, and daily 4 days exposure. At each timepoint, the exposed group was compared to its matching control group that received corn oil (380 nm kinetic measurements, total protein normalization by Bradford technique). * α = 0.05 significance level.

The measurement of GST activity performed on pulmonary membrane extracts of Helix aspersa maxima showed slight differences between snails exposed by foot injection either to 4 mg of naphthalene or corn oil (control group). Those differences in enzyme activity were modified upwards or downwards depending on the duration of exposure to the component. Indeed, a slight 25% decrease in GST activity in the pulmonary membrane was observed in snails after 24 h of naphthalene exposure compared to controls (*p*-value = 0.39; α = 0.05 significance level). The measurement of GST activity 4 days after the naphthalene injection seemed to return to baseline levels. On the contrary, extracts analyzed after 4 days of daily naphthalene exposure in similar conditions showed a 38% increase in GST activity (*p*-value = 0.017; α = 0.05 significance level). In this experiment, although a downward trend in activity can be observed during a single-dose regimen, only repeated exposure appears to significantly increase pulmonary GST activity (Figure 1).

### 3.2. ^1^H-NMR Profiles of Pulmonary Membrane After Exposure to Naphthalene

**Figure 2:**
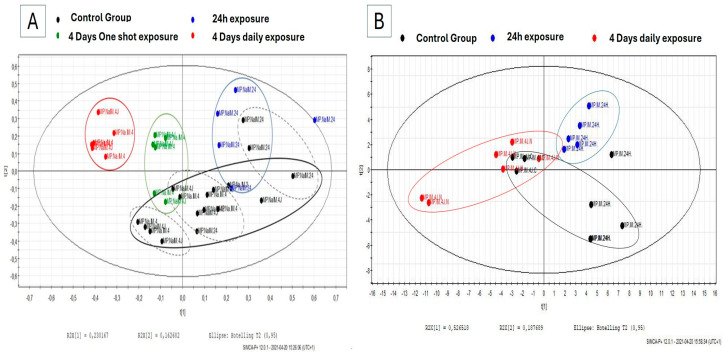
(**A**) PLS-DA score scatter plots of ^1^H-NMR binned data obtained from pulmonary membrane aqueous extracts from snails exposed to 4 mg of naphthalene (Na) for 24 h (in blue), after 4 days (in green) and after 4 days of daily exposure (in red) compared to their timely matching controls (in black, exposure times separated in black dotted line). (**B**) PLS-DA score scatter plots of ^1^H-NMR spectral data obtained from snail hemolymph exposed to 4 mg of naphthalene (Na) for 24 h (in blue) and daily 4 days (in red) compared to their timely control group exposed to corn oil (in black).

The ^1^H-NMR spectra obtained from pulmonary membrane extracts of individuals exposed to naphthalene were binned, and the numerical values retrieved after integration of the descriptor regions were submitted to multivariate data analyses and compared to controls. The PLS-DA score scatter plot reported in Figure 2 reveals some clusters of samples collected from snails exposed to 4 mg of naphthalene depending on exposure time (Figure 2A). The dispersion observed among control individuals is linked to the different regimens (duration and doses of corn oil received) to which they were submitted. Nevertheless, a clear separation between the daily naphthalene-exposed group and its corresponding control (Figure 2A) can be seen.

The metabolites contributing to the clustering were identified according to their chemical shift and the multiplicity of their corresponding resonances. Peak assignments in pulmonary membrane extracts of *Helix aspersa maxima* obtained by ^1^H-NMR spectroscopy were previously described by Devalckeneer et al. (2019) [51]. Only those discriminant metabolites displaying a VIP score ≥1 were considered for further analysis.

The changes observed in the levels of those discriminant metabolites in the pulmonary membrane tissue during exposure to naphthalene are shown in the heatmap projection constructed from the mean values of metabolite integrals presented in Table 1.

Therefore, the first metabolic changes observed after 24 h of exposure to naphthalene are lactate and succinate, which showed early increased levels followed by significant decreases 4 days after the injection. As for the daily consecutive dosing regimen, those same metabolites were maintained at lower levels as well. The opposite response was observed for ornithine, which decreased during the first 24 h and then increased when evaluated 4 days later and also in the repeated dose regimen. Glutamine and glycerol levels also increased at 24 h but seemed to return to pre-test values when assessed after 4 days. In the repeated dose regimen, glutamine level also decreased contrarily to glycerol, which kept increasing. A drop in betaine occurred after 24 h and appeared to be maintained after 4 days, whereas no change was observed with daily injections. Some modifications were only detected during this first 24 h without being changed anymore afterward, such as xanthine, sarcosine, acetyl carnitine, and glucose. Although the choline level was unchanged at first, it significantly increased after 4 days and, overall, after repeated injections.

In addition, the repeated exposure to naphthalene for 4 days showed many significant changes in the pulmonary membrane of the snail, including higher levels of fucose, acetyl-lysine, asparagine, and phenylalanine, as well as lower levels of isocitrate, malonate, glycine, UDP-glucose, and AMP.

### 3.3. ^1^H-NMR Profiles of Hemolymph After Na Exposure

^1^H-NMR spectra of the hemolymph samples for the 24 h and daily exposure groups were submitted to multivariate data analyses and compared to their matching controls. The PLS-DA score scatter plot reported in Figure 2B reveals some clusters between the two exposed groups. VIP lists were extracted from each supervised multivariate analysis, from which only those descriptors with a VIP score ≥ 1 were pinpointed and identified according to their chemical shift and multiplicity by comparison with in-house and web databases. The heatmap projection (Table 1) was constructed from mean values integrals allowed to show their levels changes in hemolymph collected from snails exposed to naphthalene as a function of exposure duration.

Decreasing levels of lactate and acetate were detected as soon as 24 h after naphthalene injection, and those decreases were even more pronounced after 4 days of exposure. The same trend, although without reaching statistical significance, was noticed for betaine and, to a lesser extent, for choline. Isoleucine and isobutyrate showed an opposite timely response to the exposure to naphthalene, such as isopropanol, which statistically increased during the first 24 h of exposure and then slightly decreased after 4 days of daily naphthalene injections. Most of the metabolite variations were observed in hemolymph after 24 h as compared to longer exposure times. For instance, it was the case for lower levels of threonine and alanine and higher levels of methanol, which were only observed at 24 h of exposure. The metabolites behind some of the modified resonances could not be identified (chemical shifts of 6.72 and 6.74 ppm in snail hemolymph) based on the sole proton NMR information.

## 4. Discussion

### 4.1. Metabolomic Evidence for Energetic Adaptation to Naphthalene Exposure

In normal conditions, snail intermediary metabolism relies on the oxidation of glucose through glycolysis, the TCA cycle, and oxidative phosphorylation. In the case of stress, exposure to naphthalene increases the body’s energy needs, pushing the glycolytic activity and resulting in an overconsumption of **glucose** and excretion of larger amounts of **lactate** [52], as observed in our experiments. When glycolysis is no longer sufficient to satisfy energy supplies, non-carbohydrate precursors such as glycerol, lactate, or even certain amino acids during gluconeogenesis take over. **Glycerol**, which is released from triglyceride lipolysis, can play a role in energy rebalancing, just like **alanine**, a glucoforming amino acid, both of which showed early changes in our study [53].

Changes in lactate from 24 h, on the other hand, could also indicate mitochondrial damage, key organelles in cell respiration. Lactate overproduction is generally associated with tissue hypoxia. These findings are supported by a study that reported **decreased glucose and increased lactate** levels in lung tissue from rats with ventilator-induced lung injury [54]. This switch to anaerobic metabolism is also supported by the concomitant **succinate** accumulation largely described in a range of tissues underexposed to oxygen [55] and described to be a conserved hallmark of ischemia in mice, rats, rabbits, pigs, and humans [56]. Indeed, succinate is involved in numerous metabolic pathways, including cellular respiration as a component of the TCA cycle, in which it is oxidized to fumarate by the enzyme succinate dehydrogenase (SDH). This dual-role enzyme, also known as complex II in the oxidative phosphorylation chain, is described as a conserved enzyme across species since it appears to play broadly similar and essential roles in all eukaryotes [57]. Interestingly, high levels of **succinate and lactate** were also observed in lung extracts by Ling et al. 2014 during intramuscular naphthalene injection in mice after 48 h [48].

Accumulation of **malonate**, the β-carbon dicarboxylic acid and a regulatory element of β-oxidation, has already been reported in case of deficiencies of the enzyme malonyl-CoA decarboxylase (MCOD), which converts malonyl-CoA to acetyl-CoA [58,59]. This acetyl-CoA deficiency, suggested by the downward trend of acetylcarnitine at 24 h of exposure [60], could impact the pyruvate dehydrogenase (PDH) activity responsible for controlling the aerobic oxidation rate of carbohydrates, thereby interfering with glucose homeostasis [61]. Such early intensive energy needs rapidly deplete the resources, as indicated 4 days later. Indeed, this failure in energy supply is supported by a higher **glycerol** synthesis, needed for energy rebalancing via lipolysis, as well as the decreased succinate level of **succinate**, the one-carbon (1C) molecule formate, and the short-chain fatty acid acetate, that can be used as a carbon source in nutrient-limited tumor cells [62,63]. **Decreasing succinate**, **acetate**, **and formate** levels were also observed in the lungs and BALF of mice after i.p. injection of naphthalene [41]. However, these metabolic modifications occurred within 24 h as compared to the present snail model. The repeated dose protocol also led to disturbances in energy metabolism as evidenced by lowered **alanine and lactate** due to gluconeogenesis activation, **succinate**, **isocitrate** through the TCA cycle, **hydroxybutyrate** through ketolysis, **malonate** through β-oxidation, and **UDP-glucose** through glycogenolysis.

### 4.2. Metabolomic Evidence for Naphthalene-Induced Oxidative Stress, Inflammatory Effects, and Immune System Response

**Choline**, a component of phospholipids, is an essential amino acid for the structural integrity of cell membranes. Phosphatidylcholine, which is composed of fatty acids and glycerol, is also considered a major lung surfactant [64]. The pulmonary surfactant system present in the gas mantel of *Helix aspersa* mainly contains phosphatidylcholine [65]. Altered lung functions due to environmental toxicants frequently show decreasing surfactant levels, such as phosphatidylcholine, in the alveolar cells after acrolein exposure [66]. Therefore, combining the changes observed in **choline and glycerol could** suggest the presence of membrane damage in lung cells.

In addition, **succinate** is known to accumulate in tissues during pathophysiological states such as acute lung injury or respiratory distress, even if its role is not well defined [67]. This accumulation, also observed after 48 h in mice treated with naphthalene [48], could be linked to alterations in SDH enzyme activity contributing to the oxidative stress via reversal electron transport [68,69,70,71], which have been recently shown to play key roles in promoting the pro-inflammatory phenotype [72], or even the oncogenesis pathway. Indeed, SDH inhibition disturbs the balance with α-ketoglutarate, playing a critical mediator role in the hypoxic response [73] by stabilization of hypoxia-inducible factors (HIFs) [74], well-known triggers of tumorigenesis [73,75,76] and described in pulmonary fibrosis [77]. The changes in **malonate**, a competitive inhibitor of SDH, support our interpretation of succinate accumulation.

Our findings also suggest different structural modifications of biomolecules. **Fucose**, a deoxy hexose involved in fucosylation, plays a critical role in cell signaling, immune response, and cell adhesion [78]. Data suggest that airway epithelial cells directly respond to allergens by increasing fucosylation [79]. **Lysine acetylation** emerged as a key regulatory mechanism, underscoring its physiological relevance in gene regulation, cell signaling, cell growth metabolism, and disease [80]. This post-translational modification of histones, recently extended to many other proteins, can modulate immune activity in various ways [81]. Therefore, increasing fucose and N-acetyl-lysine after cumulated doses of naphthalene could suggest an activation of the immune system. Other metabolic changes also support the hypothesis of a naphthalene-induced immune system response. For example, it has been demonstrated that higher production of defense cells is correlated with higher sources of **glutamine** [82], a metabolite synthesized in lungs through glutamine synthase, and consistently tends to increase across our experiments [83].

When the impact of repeated doses of naphthalene was assessed, some clues on enhanced anti-oxidative capacity were noticed. First, the modified **glycine** levels were indicative of an alteration in the biosynthesis of glutathione, a powerful antioxidant molecule. This effect was reflected in sarcosine, the methylated form of glycine synthesized through the folate cycle, and betaine, a scavenger of excess ROS and a methyl donor to generate S-adenosylmethionine (SAM). **Choline**, which also participates in methylation reactions following oxidation to betaine by the choline dehydrogenase, is considered a major source of 1C units, metabolism formed by the interconnection of methionine and folate cycles. These pathways, conserved from yeast to mammals [84], are known to support multiple physiological processes, including biosynthesis (purines and thymidine), amino acid homeostasis (glycine, serine, and methionine), epigenetic maintenance, and redox defense [85].

As a precursor of putrescine following its decarboxylation, **ornithine** is involved in the polyamine pathway recognized for its crucial role in ROS homeostasis [86]. Moreover, changes in **inosine**, a purine nucleoside involved in adenosine metabolism, indicate a higher polyamine synthesis but a slowdown in glycolysis [87]. Interestingly, inosine has already been reported for its anti-inflammatory properties [88,89], especially in lung inflammation [90].

Most of those metabolic changes were seen in the pulmonary membrane extract compared to a few metabolites emerging in hemolymph, such as isopropanol, lactate, and acetate. After cumulated doses, changes in isoleucine, a branched-chain amino acid (BCAA) and nitrogen donor, could reflect a higher rate of protein biosynthesis and nucleic acid production, respectively. Short-chain fatty acids (SCFAs) such as isobutyrate are known to reduce inflammatory risk by direct interactions with the immune system via responsible receptors [91]. These various mechanisms of action can reduce the number and migration ability of immune cells [92,93].

**Figure 3:**
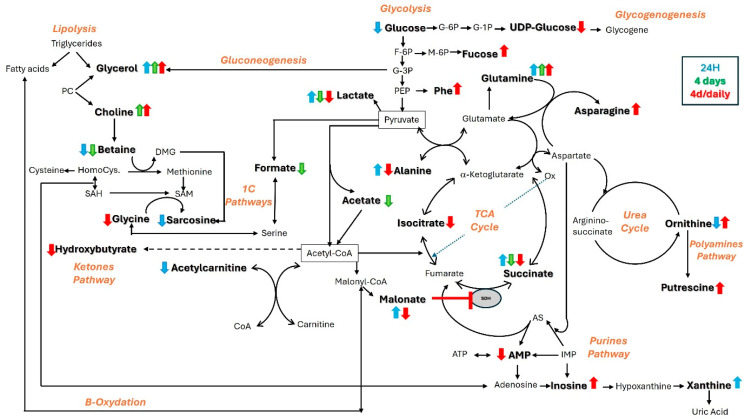
Alterations in metabolic pathways in tissue extracts of pulmonary membrane in *Helix aspersa maxima* following exposure to naphthalene, after 24 h (in blue), after 4 days (in green), and after 4 days of daily injections (in red). Ox: oxaloacetate, SAM: S-adenosylmethionine, SAH: S-adenosylhomocysteine, Homocys.: homocysteine, DMG: dimethylglycine, PC: phosphatidylcholine, AS: adenylosuccinate, SDH: succinate dehydrogenase.

**In conclusion** (Figure 3), the results of this study suggest that the global energy metabolism of snails adapts to naphthalene exposure by switching to anaerobic metabolism following possible hypoxia after 24 h of exposure to naphthalene. After four days of exposure, this energy metabolism appears to be exhausted, and oxidative stress and membrane damage occur. Cellular damage caused by oxidative stress is a well-known mechanism of naphthalene toxicity following its metabolism into reactive compounds. In addition, our findings support a possible alteration in purine metabolism as well as an activation of the immune system via the promotion of defense cells fueled by 1C and glutamine metabolisms in response to stress.

### 4.3. A Tug of War for Nutrients in Highly Proliferative Cells?

Although the primary objective of this study was the evaluation of respiratory effects in snails exposed to naphthalene, it appeared that many changes could be associated with the support of the important metabolic activity of highly proliferative cells. In fact, both immune and tumorigenic cells seem to use the same nutrient metabolic pathways, leading to the questioning of the possible dual interpretation of our metabolomic findings.

As a matter of fact, the demands in 1C metabolism promoting defense cells could also provide essential nutrients for the tumorigenic microenvironment [94,95]. Indeed, cell proliferation of tumor growth leads to a depletion of endogenous sources of single-carbon units, which support nucleotide biosynthesis [95,96,97,98]. So, tumor progression is characterized by abnormal levels of choline-containing compounds [99]. Moreover, many metabolites have been discovered in the lung tumorigenic process, including lower levels of formate, sarcosine, and glutamate [95], and a concomitant increase in glutamine and asparagine [100,101,102] due to the upregulation of asparagine synthase [101] or an increase in ornithine produced from the hydrolysis of arginine during the urea cycle [103]. The supply of arginine in the tumor environment is the subject of competition between immune and tumor cells; the latter increases its catabolism in ornithine to the benefit of their proliferation [104]. Indeed, studies have revealed that increased polyamine levels can also attenuate anti-tumor functions of immune cells to the benefit of tumor cells [105]. Likewise, inosine, which limits the immune cell activity versus tumorigenic cells [106,107], has been reported in high levels in patients with lung squamous cell carcinoma [108]. Emerging research in glycomics has again highlighted that the tumorigenic phenotype often exhibits abnormal glycosylation [109], including fucosylation [110], as suggested by a high requirement of fucose in lung tumors [111,112,113]. In the same way, proteins from post-translational modifications (PTM), including lysine-acetylation [114], support this phenotype whether via high aberrant histone acetylation, which can deregulate chromatin-based processes or via that of non-histone proteins, especially transcription factors [115,116,117]. Perturbations in BCAA blood concentrations are also a hallmark of tumor growth, as malignant cells rely on their release through protein breakdown for proliferation. Changes in isopropanol, glycerol, and isoleucine detected in the hemolymph of naphthalene-exposed snails were also reported in plasma of lung cancer patients [118,119], isoleucine, especially, positively correlated with the squamous cell lung cancer risk [120].

Therefore, future studies should imply additional indicators of tumorigenesis to validate the link between the early metabolic markers observed in this study and tumor development in this invertebrate model.

### 4.4. GST Analysis in Pulmonary Membrane

Glutathione (GSH) is the major non-enzymatic antioxidant in animal cells, involved in the protective metabolism of cells against endogenous and exogenous compounds. In addition to its role as a reducing agent, it is essential for phase II conjugation detoxification reactions catalyzed by GST.

Many publications have reported an airway epithelial tolerance with repeated exposures to naphthalene, either by inhalation or intraperitoneal injection, generally after seven days in mice [121,122]. This naphthalene tolerance, specific to the respiratory tract, is declared by an injury resistance through a significant elevation in GSH levels in lung-protected tissues compared to its depletion observed when cell damage occurred [123,124]. GST plays a key role in detoxifying the cytotoxic effects of activated naphthalene metabolites during phase I; this GSH-dependent tolerance likely proceeds via GST catalysis [125]. Indeed, studies have revealed that the expression of GST isoenzymes in the lungs of naphthalene-tolerant mice differed from controls [43].

In this study, decreased GST activity observed after 24 h of naphthalene exposure suggests either that GSH is not sufficient anymore to sustain the catalytic reaction of GST conjugation or a stock depletion of GSH used for the detoxification mechanism within the first 24 h. A decrease in GSH leading to reduced GST activity was already observed with acute exposures in lung bronchial cells and mice, but in the case of iron nanoparticle treatment [126]. Conversely, the high activity detected after cumulative naphthalene injections suggests an enhanced detoxification process against pro-oxidative forces and tissue protection from injuries. Nevertheless, studies on mice have revealed that hepatic GST was essentially responsible for naphthalene tolerance compared to its pulmonary contribution, which was not highly increased.

## 5. Conclusions

Numerous biomarkers involved in lung injury in mammal studies were detected in snails exposed to naphthalene, especially with daily exposure. Many of them could suggest inflammatory or tumorigenic pathways via nutrient sources for proliferative cells and impairment of membrane integrity. GST activity in the pulmonary membrane suggested a stock depletion of GSH in the first 24 h reaction as a detoxification process to an enhanced activity with repeated exposures implying resynthesis of GSH to protective reaction. In contrast, GSH or its precursors did not clearly show any implication, at least using the ^1^H-NMR technique.

Although early effects of naphthalene toxicity on the snail model could be observed in this study, hemolymph does not seem as relevant as lung tissue, the target of toxicity, during ^1^H-NMR analysis. However, the ease of use, including dissection, of this animal model compared to mammals, limited by animal ethics, would therefore meet the 3Rs law, especially during in situ surveillance.

In conclusion, this study demonstrates the potential of an approach combining a bioindicator invertebrate model, herein the *Helix aspersa maxima* snail, with an “omics” tool in prospective risk assessment, which could offer new research opportunities to preserve human, animal, and environmental health closely connected according to the “One Health Concept”.

## 6. Study Limitations and Perspectives

This preliminary study, designed as a proof-of-concept exploration of the metabolomic response of possible respiratory effects in a pulmonated invertebrate, represents a first step in the development of future metabolic biomarkers of toxicity which could be transposed from invertebrates to mammals, including humans.Clearly, some adjustments are needed, particularly at the statistical level, where a larger sample size should be used either to refine non-statistical metabolic changes extracted via VIPs or when quantitative dosages of the metabolites of interest will be assessed in future research. As for the use of ^1^H-NMR spectroscopy as the main analytical technique, it suffers from poor sensitivity, is insufficient for the identification of unknown resonances, and is definitely unsuitable for the observation of macromolecules. In such cases, mass spectrometry and 2D-NMR would be more suitable tools. Finally, the final interest would imply the in situ exposure of the model, which allows the exposure routes and the bioavailability of pollutants in soils to be taken into account.

Although this study was carried out in a laboratory environment as a proof of concept, it is clear that placing invertebrate individuals directly on site would allow a study closer to reality in terms of exposure routes and doses.

## Figures and Tables

**Table 1:** Heatmaps of discriminant metabolites identified by chemical shifts (in ppm) in pulmonary tissue and hemolymph samples with either increased percentage levels (redder tone) or decreased percentage levels (greener tone) in naphthalene-exposed groups after 24 h, after 4 days, and after 4 days of daily exposure as compared to their matching controls. Significant changes are displayed in italicized bold underlined characters (Wilcoxon test, α < 0.05).

In Pulmonary Tissue						In Hemolymph				
ppm	Metabolites	CTRL	After 24 H	After 4 Days	4 Days/Daily	ppm	Metabolites	CTRL	After 24 H	4 Days/Daily
1.2	Hydroxybutyrate	100			44	1–1.01	Isoleucine	100	75	204
1.24	Fucose	100			***205***	1.08–1.1	Isobutyrate	100	119	41
1.32	Lactate	100	153	***47***	***37***	1.17–1.18	Isopropanol	100	***155***	81
1.48	Alanine	100	119		69	1.28–1.29	Threonine	100	88	
1.72–1.76	Putrescine	100			168	1.17–1.20	Ethanol	100		84
1.78	Ornithine	100	65			1.33–1.34	Lactate	100	50	***23***
1.92	Acetate	100	106	50	107	1.45–1.47	Alanine	100	87	
2.03	N-acetyl-Lysine	100			***153***	1.92	Acetate	100	43	***13***
2.11–2.18	Glutamine	100	167	123	124	3.23	Choline	100	89	68
2.34–2.37	Glutamate	100	110	83	92	3.36	Methanol	100	***179***	
2.41	Succinate	100	197	***30***	***18***	3.55–3.58	Glycerol	100	126	113
2.54	Isocitrate	100			***27***	3.91	Betaine	100	17	35
2.71	Dimethylamine	100	90			6.72	Unknown	100	***119***	
2.73	Sarcosine	100	57			6.74	Unknown	100	***118***	
2.8	Aspartate	100			112	7.01	Unknown	100	51	
2.86	Dimethylformamide	100			142	7.72	Unknown	100	***44***	
2.88	Asparagine	100			***300***	7.76	Unknown	100	50	
3.04	Ornithine	100		122	***156***					
3.1	Malonate	100	132		***50***					
3.19	Acetylcarnitine	100	67							
3.21	Choline	100	92	***119***	***134***					
3.23	Acetylcholine	100	89	84	99					
3.24	Carnitine	100	109	102						
3.27	Betaine	100	83	86						
3.56	Glycine	100	101		***59***					
3.65	Glycerol	100	182	145	***303***					
3.8	Glucose	100	79							
5.97	UDP-Glucose	100			***43***					
6.08	Adenosine	100			84					
6.1	Inosine	100		123	198					
6.15	AMP	100			***20***					
6.52	Fumarate	100	114	101						
6.88	Tyrosine	100	92							
7.33	Phenylalanine	100			***204***					
7.69	Unkown	100		***281***	125					
7.88	Uridine	100	98							
7.9	Xanthine	100	192							
8	Guanosine	100			91					
8.46	Formate	100		37						
	Level modifications of metabolites									
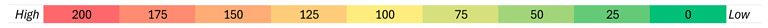										

## Data Availability

The original contributions presented in this study are included in the article. Further inquiries can be directed to the corresponding author(s).

## Abbreviations

The following abbreviations are used in this manuscript:

PAH	Polycyclic aromatic hydrocarbon
TCA	Tricarboxylic acid cycle
BALF	Bronchial alveolar lavage fluid
GST	Glutathione S-transferase
GSH	Glutathione
CDNB	1-chloro-2,4-dinitrobenzene
BSA	Bovine serum albumin
D_2_O	Deuterium oxide
TSP	D_4_-trimethylsilyl propionic acid
FID	Free induction decay
AUC	Area under the curve
PLS-DA	Partial least squares discriminant analysis
VIP	Variable importance in projection
AMP	Adenosine monophosphate
ATP	Adenosine triphosphate
MCOD	Malonyl-CoA decarboxylase
PDH	Pyruvate dehydrogenase
i.p.	Intraperitoneal
SDH	Succinate dehydrogenase
HIF	Hypoxia-inducible factor
ROS	Reactive oxygen species
SAM	S-adenosylmethionine
PTM	Post-translational modification
BCAA	Branched-chain amino acid
SCFA	Short-chain fatty acid
